# On Lateness: The Ethics of Running Behind Schedule in General Practice

**DOI:** 10.1111/jep.14293

**Published:** 2024-12-23

**Authors:** Richard C. Armitage

**Affiliations:** ^1^ Academic Unit of Population and Lifespan Sciences, School of Medicine University of Nottingham, Clinical Sciences Building, Nottingham City Hospital Campus Nottingham UK

**Keywords:** medical ethics, primary care, professionalism

## Abstract

**Introduction:**

GPs, at least in the United Kingdom, often run behind schedule in their clinics. This lateness is an inherently ethical problem due to the negative consequences it generates.

**Methods:**

The paper outlines these negative consequences, attempts to classify the major reasons for such lateness, explores the ethical status of each of these reasons, and offers suggestions for how the negative consequences might be managed.

**Findings and Discussion:**

The major reasons for lateness can be classified as GP‐related, patient‐related, and third party‐related. The major negative consequences of lateness in general practice might be classified as the potential disturbance to quality and safe care, the dissatisfaction of and inconvenience to subsequent patients, and the disruption of timely care. These negative consequences must be burdened by some party—either the patient who is related to the reason for the lateness, or other patients who are not. While a strict equality approach to managing such lateness does not consider patients’ clinical needs, GPs compensating by actively ‘catching up’ in their clinics threatens quality and safety of care. The paper argues for minimising the negative consequences of lateness for all parties, while simultaneously promoting equity with regard to patients’ clinical needs. The ethical status of each major reason for lateness in general practice is explored, and suggestions are offered for how each might be managed to minimise the negative consequences and promote equity.

## 1 Introduction

Routine general practice in the United Kingdom largely involves general practitioners (GPs) conducting clinics of individual patient consultations in their surgeries. Each consultation is allocated a 10‐ or 15‐min appointment, which is booked in advanced and scheduled to begin at a pre‐specified time. Each GP consults with 37 patients per day on average [1] in addition to, amongst other activities, the associated administrative burden of patient consultations, supporting general practice colleagues in their clinical practice, and communicating with colleagues in secondary care.

At least in the United Kingdom, GPs have long been recognised to often run behind schedule in their clinics [2]. The prevalence of, and the disruption caused by, this lateness is such that both GPs and GP surgeries make efforts to explain why such lateness frequently occurs [3, 4, 5, 6, 7].

Lateness in general practice is an inherently ethical problem as a direct result of its consequences. This paper shall outline these consequences, attempt to classify the major reasons for such lateness, explore the ethical status of each of these reasons, and offer suggestions for how the negative consequences might be managed.

## 2 The Consequences of Lateness

The negative consequences of GPs running behind schedule might be classified into three major groups: first, lateness might impede the delivery of quality and safe care. This is because GPs might feel pressure to ‘catch up’ in their clinics and compensate for their lateness to minimise its impact on subsequent patients. This can only be achieved by condensing the time spent in those subsequent consultations until the GP is once again running on time. This shortening of consultation time risks reducing both the quality and safety of the care provided in those consultations (the perception of time pressure, or time shortage, has long been recognised as a strong risk factor for medical error) [8]. This might result from, for example, the GP failing to gather all relevant information, overlooking important factors, or communicating with patients in a suboptimal manner (the strong association between GP communication and patient satisfaction has long been recognised) [9].

Second, lateness might inconvenience subsequent patients in the GP's clinic and reduce patient satisfaction. GPs running behind schedule requires those patients who arrive on time for their appointments to spend unexpected time in the waiting room, which might disrupt their subsequent plans. Such unexpected waiting in general practice is associated with lower patient satisfaction [10, 11], and doctor lateness in other clinical specialties has been shown to generate feelings of unimportance in the affected patients and the perception of disrespect for their time [12]. If the GP is running substantially behind schedule, it is possible that patients who are themselves on a strict agenda might abandon the waiting room and forego their appointment and, therefore, not receive the care they hoped to receive, which is likely to reduce patient satisfaction.

Third, lateness might impede the timely provision of care. While most patient consultations in general practice do not involve the delivery of time‐critical care (such severely ill patients almost exclusively access care directly via emergency ambulance or Emergency Departments), clinical emergencies do occur in general practice [13]. Examples include presentations of acute chest pain, severe shortness of breath, non‐blanching rash, hypoglycaemia, anaphylaxis, and seizures [14]. The potentially life‐threatening nature of these presentations requires their immediate clinical attention. In general practice, this usually involves the administration of initial treatments (such as high‐dose aspirin, high‐flow oxygen, intramuscular antibiotics, or intramuscular adrenaline) [14], calling for an emergency ambulance, and monitoring the patient before the paramedics’ arrival. If the GP is unaware of the clinical urgency of such presentations (such as if the reception staff do not recognise the patient's appearance as being worrisome, or if their concern is not communicated to the GP), the patient's clinical care will be delayed due to the GP's lateness. In life‐threatening situations, this delay in the provision of care might lead to poorer patient outcomes, in addition to the prolongation of the patient's suffering (such as pain and shortness of breath).

## 3 Major Reasons for Lateness in General Practice

The reasons for why GPs run behind schedule might be classified into three major groups: those related to the GP, those related to the patient, and those related to third parties.

### 3.1 GP‐Related Reasons

There appears to be one main GP‐related reason for why GPs run behind schedule: arrival delays, such as if the GP encounters heavy traffic or challenging weather conditions while travelling to the GP surgery.

### 3.2 Patient‐Related Reasons

Six main patient‐related reasons for why GPs run behind schedule might be identified: first, arrival delays, such as those due to heavy traffic or challenging weather conditions, or if the patient's appointment is located at a facility they are unfamiliar with, such as a branch or satellite of the primary GP surgery; second, lateness that is secondary to late‐arriving patients, such as if a patient arrives 9 min late for a 15‐min appointment; third, consultations with patients who have complex medical histories (such as multimorbidity, polypharmacy, and frailty), challenging social factors, or multiple complaints brought to single appointments, or a combination of these; fourth, communication barriers, such as when the patient does not speak the same language as the GP with the competence necessary for quality and safe care, and therefore requires the use of an interpreter; fifth, clinical emergencies in the GP's clinic, which require arrangement of specialist assessment (such as Mental Health Crisis Intervention Team for suicidal or patients with acute psychosis) or hospital admission arrangement (including calling for an emergency ambulance, communicating with secondary care colleagues, and writing referral letters), administering emergency treatment, and monitoring the patient until hospital transfer begins); sixth, children who are brought to their appointment late by their parent or guardian.

### 3.3 Third Party‐Related Reasons

Five main third party‐related reasons for why GPs run behind schedule might be identified: first, practical problems, such as IT failures (e.g., freezing computers, electronic health record outages, and printer malfunctions) [15], power outages, or fire alarms requiring building evacuation; second, contacting secondary care colleagues, such as when the GP calls a specialist for advice and must wait a substantial amount of time to reach them, and spend further time communicating the problem and receiving the response; third, interruptions by colleagues, such as when another clinician in the GP surgery requests a second opinion, clinical advice, support with an IT matter (such as navigating local online referral pathways, electronic health records, and online management protocols), and assistance with the management of a clinical emergency in their own clinic; fourth, a clinical emergency in the GP surgery that does not occur in the GP's own clinic, or in any other clinician's clinic, such as a patient collapsing in the waiting room, falling in the carpark, or presenting with acute chest pain without a pre‐booked appointment; fifth, errors by colleagues, such as if a patient arrives on time but is not recorded as having arrived on the electronic appointment programme such that the GP is unaware of the patient's presence in the waiting room, or if the patient's electronic health record has not been adequately shared between the relevant organisations such that the GP does not immediately have the required access to provide quality and safe care.

## 4 The Ethical Status of Reasons for Lateness

Lateness in general practice is an ethical problem because its negative consequences must be burdened by some party. The major question for practical ethics is *which* party should burden these consequences—the patient who is related to the reason for the lateness, or other patients who are not?

A strict equality approach to this question would entitle each patient to no more of the GP's time than that bounded by their pre‐scheduled appointment time and duration, such as 10 min between 11:00 and 11:10. For example, if the patient arrives at 11:07, they can consult with the GP for no more than 3 min and, if they arrive at 11:11, they cannot consult at all. This ensures that the negative consequences of the lateness are borne exclusively by the patient who is related to the reason for it. However, this seems unfair for at least three reasons: first, it is unlikely that quality care that leaves the patient satisfied can be safely delivered within 3 min; second, it does not consider the reason for the lateness, which might lie entirely outside the patient's control; third, it does not consider the patient's clinical need, which might outweigh the negative consequences of the lateness, even if they are borne by other patients.

An alternative approach is for the GP to actively try to ‘catch up’ in their clinic. This might require the GP to take ‘shortcuts’ to reduce the length of subsequent consultations, which is likely to be detrimental to quality and safety of care, and to reduce patient satisfaction. Therefore, while naturally brief consultations (such as simple medication reviews) might passively allow the GP to catch up, it is not ethically acceptable for the GP to actively shorten consultations to compensate for lateness.

A more ethically permissible approach, therefore, might be to minimise the negative consequences of lateness for both the patient who is related to the reason for the lateness, and other patients who are not, while simultaneously promoting equity with regard to clinical need. In practice, GP surgeries often attempt this through implementation of a time‐bound grace period in which late‐arriving patients are guaranteed to consult with the GP up to a certain degree of lateness. For example, patients who arrive less than 10 min late for their 15‐min appointment will still consult, and whether those who arrive later will consult or not is subject to the GP's discretion. This discretion allows relevant variables to be considered, such as the patient's clinical need (e.g., an emergency appointment booked for shortness of breath suggests an urgent clinical need, while a routine annual review does not).

The ethical status of each major reason for lateness in general practice shall now be explored, and suggestions made for how the negative consequences of each might be minimised and equity regarding clinical need promoted.

### 4.1 GP‐Related Reasons: Arrival Delays

Punctuality is a tenant of professionalism across all industries. GPs are expected by both patients and colleagues to arrive at the workplace in good time to begin their clinics in a prepared and prompt manner. While occasional arrival delays due to unexpected events are inherently unavoidable, frequent arrival delays are preventable, and are therefore both unprofessional and ethically impermissible, since the negative consequences of this lateness are necessarily borne by the GP's patients. GPs, therefore, ought to take sufficient steps to ensure that arrival delays rarely occur, such as by ensuring that the timing of their commute considers potential heavy traffic and challenging weather conditions.

### 4.2 Patient‐Related Reasons: Arrival Delays

Unlike GPs, there is no professional expectation for patients to be punctual for their appointments, yet the negative consequences of lateness generate a social expectation of patient punctuality. While infrequent episodes of arrival delay due to unexpected events are unavoidable, and lateness due to unfamiliarity with the whereabouts of a branch surgery is understandable, recurrent arrival delays are less acceptable due to the repeated negative consequences they generate. GP surgeries, therefore, ought to communicate the importance of punctuality, and might consider a time‐bound grace period (in which the GP is able to override its limits at their discretion), to prevent this.

### 4.3 Patient‐Related Reasons: Lateness Secondary to Late‐Arriving Patients

A patient who arrives, for example, 9 min late for their 15‐min appointment, and requires 15 min for the delivery of quality and safe care, will cause the GP to begin consulting with their next patient at least 9 min late. Unless the GP ‘catches up’ on their schedule (which is ethically unacceptable when actively pursued), cumulative lateness could propagate through the clinic, the negative consequences of which are borne by multiple subsequent patients. Again, GP surgeries ought to communicate with their patients the importance of punctuality for GP appointments, and might consider a time‐bound grace period (with GP discretion) to limit cumulative lateness.

### 4.4 Patient‐Related Reasons: Complexity

This might be the most prevalent reason for lateness in general practice. The complexity of consultations (which includes, amongst other factors, multimorbidity, polypharmacy, frailty, medically unexplained symptoms, terminal diagnoses, mental health problems, dementia, problematic drug and alcohol use, and challenging social circumstances such as homelessness, safeguarding concerns, and immigration status) [16] is increasing [17, 18]. In addition, GPs often deal with multiple independent problems in each appointment [19, 20], which is likely to increase as patients experience long waits for appointments and ‘save up’ various problems for single consultations [21]. This combination is likely to prevent consultations from being completed within their allocated time, thereby placing the GP behind schedule. Such lateness caused by complexity might be ethically permissible because the patients’ clinical needs are such that they necessitate consultations that exceed the standard duration. The resulting negative consequences burdened by subsequent patients are unlikely to outweigh the benefit afforded to the patient by their clinical needs being met, but not beyond a certain degree of lateness. If the resulting lateness is substantial, the negative consequences of recurrent episodes might be avoided by such patients being granted double‐appointments, although this raises the further ethical problem of scarce resource allocation. Lateness caused by multiple problems per appointment might also be ethically permissible because it avoids the need for additional appointments, thereby preserving them for use by other patients. However, multiple problems condense the time that can be dedicated to each, which threatens both the quality and safety of the care provided to them, while the resulting lateness imposes its inherent negative consequences on subsequent patients. ‘One problem per appointment’ policies, which refer to ‘major’ problems and allow GPs to deal with additional ‘minor’ problems at their discretion, is one way to address this and have been implemented by various GP surgeries [22, 23, 24].

### 4.5 Patient‐Related Reasons: Communication Barriers

A substantial proportion of individuals living in the United Kingdom lack sufficient command of English to allow direct communication with their GP: in 2021, 5.1 million people in England and Wales (8.9% of the total population aged 3 and over) did not consider English (or Welsh in Wales) as a main language and, of these people, 1,041,000 could either not speak English well (880,000) or could not speak English at all (161,000) [25]; in 2021, 20,200 people in Northern Ireland (1.1% of the total population aged 3 and over) could not speak English well or not at all [26]; and, in 2022, 60,862 people in Scotland (1.1% of the total population aged 3 and over) had limited (51,795) or no skills in English (9067) [27]. An interpreter is required to facilitate a consultation if the patient is unable to speak the same language as the GP with sufficient competence to allow quality and safe care. Casual interpreters, such as family members or friends, might be brought to consultations, but professional interpreters are strongly preferred [28], and are always necessary in the absence of casual interpreters or if the casual interpreter is a child [29]. While professional interpreters can be pre‐arranged (and physically accompany the patient to the appointment), they might also be accessed on a reactive basis during consultations via telephone interpretation services. Consultations facilitated by interpreters take at least twice as long as those without, since each question and answer must be stated twice (once in each language) [25]. Additional time is also required to access professional interpreters via telephone interpretation services, especially if the required language is infrequently spoken such that the availability of relevant interpreters is limited. Such consultations are likely to place the GP behind schedule. However, interpreters are essential for the provision of quality and safe care, and are thus indispensable for equity regarding clinical need. The negative consequences of the resulting lateness might be minimised by pre‐arranging interpreters (especially for infrequently spoken languages), and double‐appointments being allocated to patients who require them (although this raises the ethical problem of scarce resource allocation).

### 4.6 Patient‐Related Reasons: Clinical Emergencies in the GP's Clinic

If the GP considers a patient in their clinic to be in a state of clinical emergency, immediate action is necessary. A mental health crisis, such as suicidality or acute psychosis, requires immediate referral to the local Mental Health Crisis Intervention Team, which often involves a lengthy telephone conversation. A medical or surgical emergency requires admission to hospital (which might involve the GP calling an emergency ambulance, communicating with secondary care colleagues via telephone, and writing referral letters), administering emergency treatment, and monitoring the patient before the paramedics' arrival. Emergency situations are highly likely to place the GP behind schedule, yet the negative of consequences of this lateness are outweighed by the benefit afforded to the subject of the emergency, who might otherwise experience profoundly negative health outcomes (or even death) without receiving emergency care. In such situations, the clinical need of the emergency overrides the negative consequences of the resulting lateness that is burdened by subsequent patients, and the GP is both professionally and ethically obligated to wholly attend to the emergency.

### 4.7 Patient‐Related Reasons: Children Brought Late

Children brought to their appointment late by their parent or guardian could not have done otherwise. It seems unfair, therefore, that they should burden the consequences of this lateness for which they are not responsible (such as having a shortened consultation, or no consultation whatsoever). Accordingly, GPs ought to allow substantial leniency over any individual breach of a time‐bound grace period when the patient is a child. However, recurrent instances are less acceptable due to the repeated negative consequences they generate, and ought to trigger GP surgeries to communicate the importance of punctuality to the parent or guardian.

### 4.8 Third Party‐Related Reasons: Practical Problems

The occurrence of practical problems, such as IT failures, power outages, and fire alarms, can place the GP substantially behind schedule. Fire alarms must be responded to with immediate building evacuation by both patients and staff as they alert those within it to a dangerous hazard. The resulting lateness is therefore acceptable. Power outages deny access to electronic health records and other resources that are necessary to deliver quality and safe care, so the resulting lateness is also acceptable. While IT failures are largely unavoidable, they can be somewhat mitigated against by the regular maintenance and upgrading of these technologies, which GP surgeries ought to invest in to, amongst other reasons, minimise the negative effects of the lateness their failures cause.

### 4.9 Third Party‐Related Reasons: Contacting Secondary Care Colleagues

Secondary care facilities often contain colleagues in each clinical specialty who are designated to receive telephone calls from GPs in need of specialist advice. These colleagues generally take such calls in addition to their own clinical workload (such as during operating lists), meaning GPs must sometimes wait substantial amounts of time before being connected with them. Further time is then spent communicating the nature of the problem, the colleague reviewing any relevant secondary care records, and the specialist advice being conveyed to the GP. Such delays are likely to place the GP behind schedule, yet this is acceptable since such immediate specialist advice is required for quality and safe care. This lateness, and the resulting negative consequences, could to be minimised by secondary care colleagues being allocated a lighter clinical workload when they are designated to receive GP calls.

### 4.10 Third Party‐Related Reasons: Interruptions by Colleagues

GPs might be asked to assist colleagues in the management of clinical emergencies in their own clinics. This is highly likely to place the GP behind schedule. As when a GP encounters an emergency in their own clinic, the clinical need of the emergency overrides the negative consequences of the resulting lateness, and the GP is obligated to assist. GPs might also be asked by colleagues for second opinions, clinical advice, and support with IT matters to facilitate the care of patients in their own clinics. Individual instances of such interruptions are acceptable as they promote the quality and safety of care provided to those patients, but recurrent interruptions are less acceptable due to the repeated negative consequences of the lateness they generate. GP surgeries might designate a specific GP to deal with such queries each day, and allocate them a lighter clinical workload during these periods.

### 4.11 Third Party‐Related Reasons: Clinical Emergencies Located Elsewhere

Clinical emergencies might also occur outside the GP's clinic, and outside the clinic of all other clinicians, such as in the GP surgery's waiting area, bathroom or carpark. The subjects of these emergencies might be patients who are attending the surgery for appointments, those accompanying such patients, or other individuals who present without pre‐booked appointments. As with emergencies in the clinics of the GP and other clinicians, the GP is obligated to assist with these emergencies, as the clinical need of the subject overrides the negative consequences of the resulting lateness. This is regardless of which clinic, if any, the patient is attending.

### 4.12 Third Party‐Related Reasons: Errors by Colleagues

Errors by colleagues, such as reception staff failing to record a patient as having arrived, or not appropriately sharing electronic health records, might place the GP behind schedule. This is because it would be unfair for the patient in question to burden the consequences of the colleague's error, meaning the patient must still consult with the GP once the error has been identified and corrected. The resulting lateness generates negative consequences that must instead be borne by subsequent patients. GP surgeries ought to minimise these consequences by providing staff with sufficient and regular training to prevent such errors.

## 5 Conclusion

GPs often run behind schedule in their clinics, the reasons for which can be classified as GP‐related, patient‐related, and third party‐related. The major negative consequences of lateness in general practice can be classified as the potential disturbance to quality and safe care, the dissatisfaction of and inconvenience to subsequent patients, and the disruption of timely care. These negative consequences must be burdened by some party—either the patient who is related to the reason for the lateness, or other patients who are not. While strict equality applied to lateness does not consider patients’ clinical needs, GPs actively ‘catching up’ threatens quality and safety of care. This paper argues for minimising the negative consequences of lateness for all parties, while simultaneously promoting equity regarding clinical need, and offers suggestions for how this might be achieved.

## Conflicts of Interest

The author declares no conflicts of interest.

## Data Availability

The author has nothing to report.
